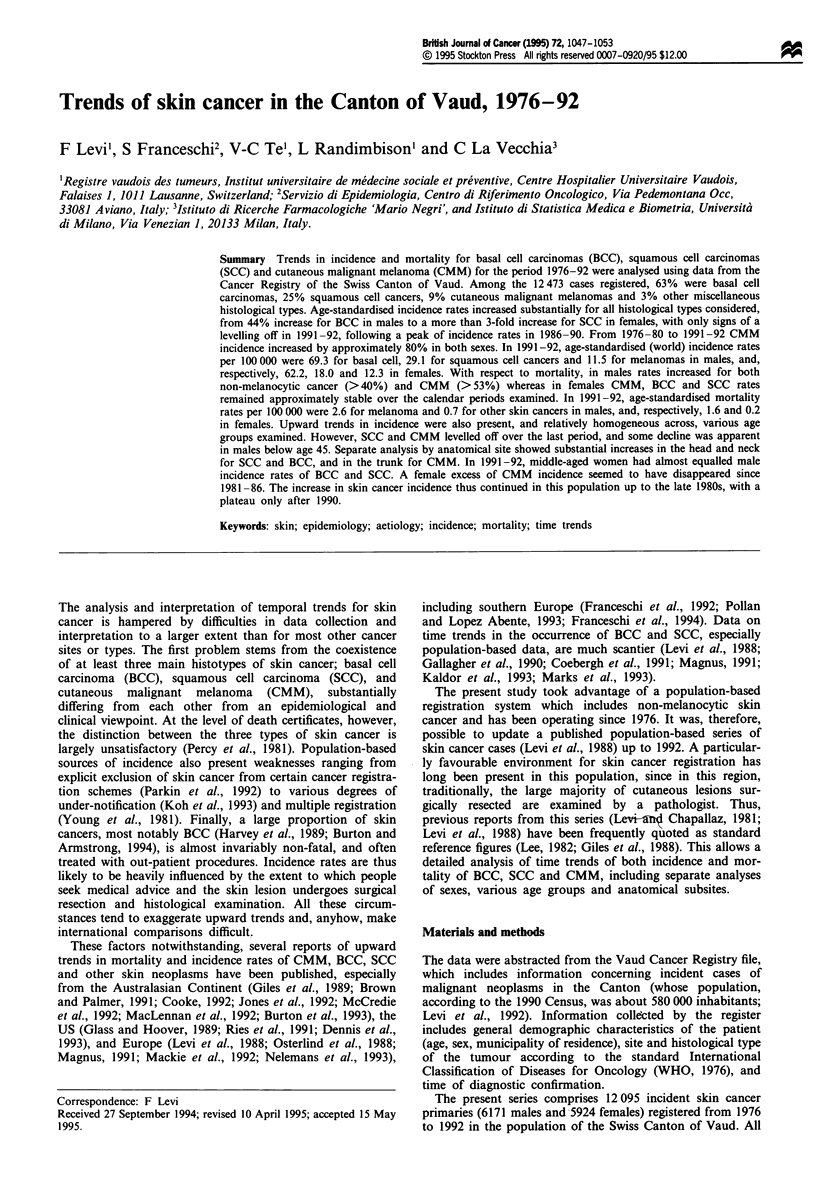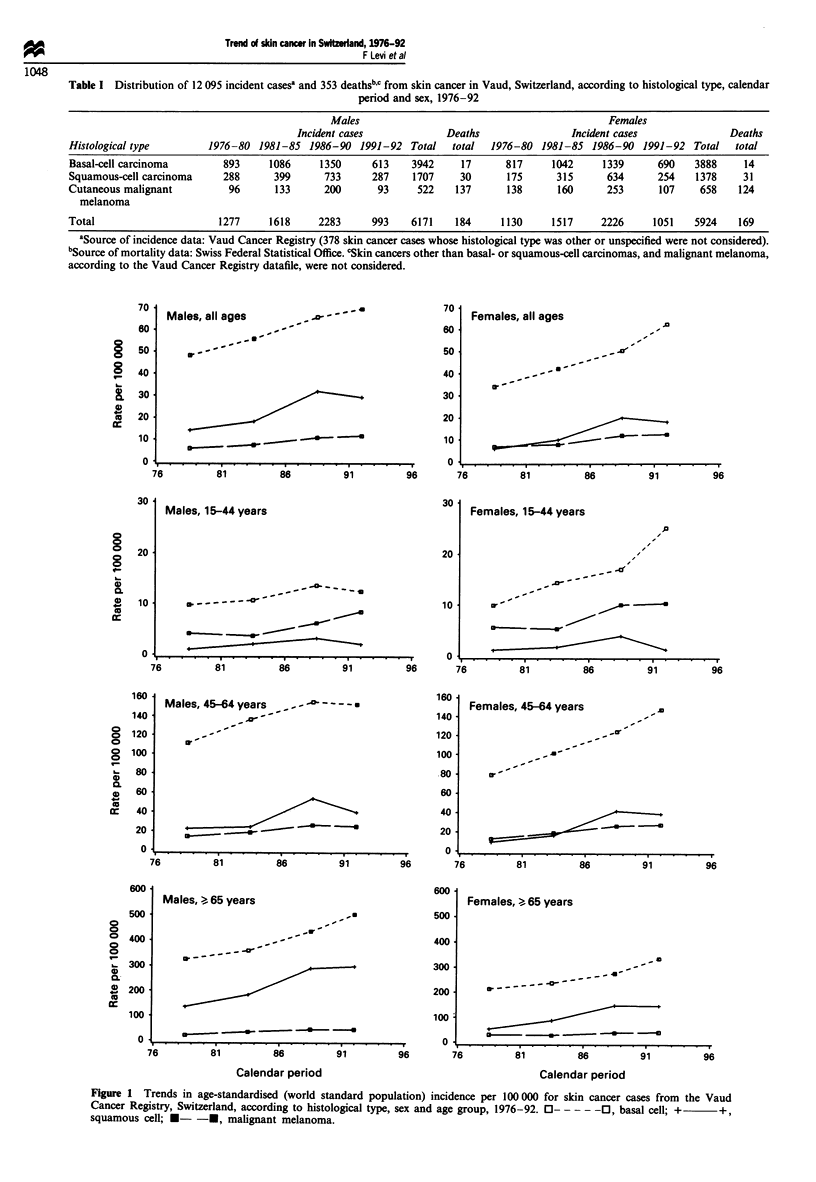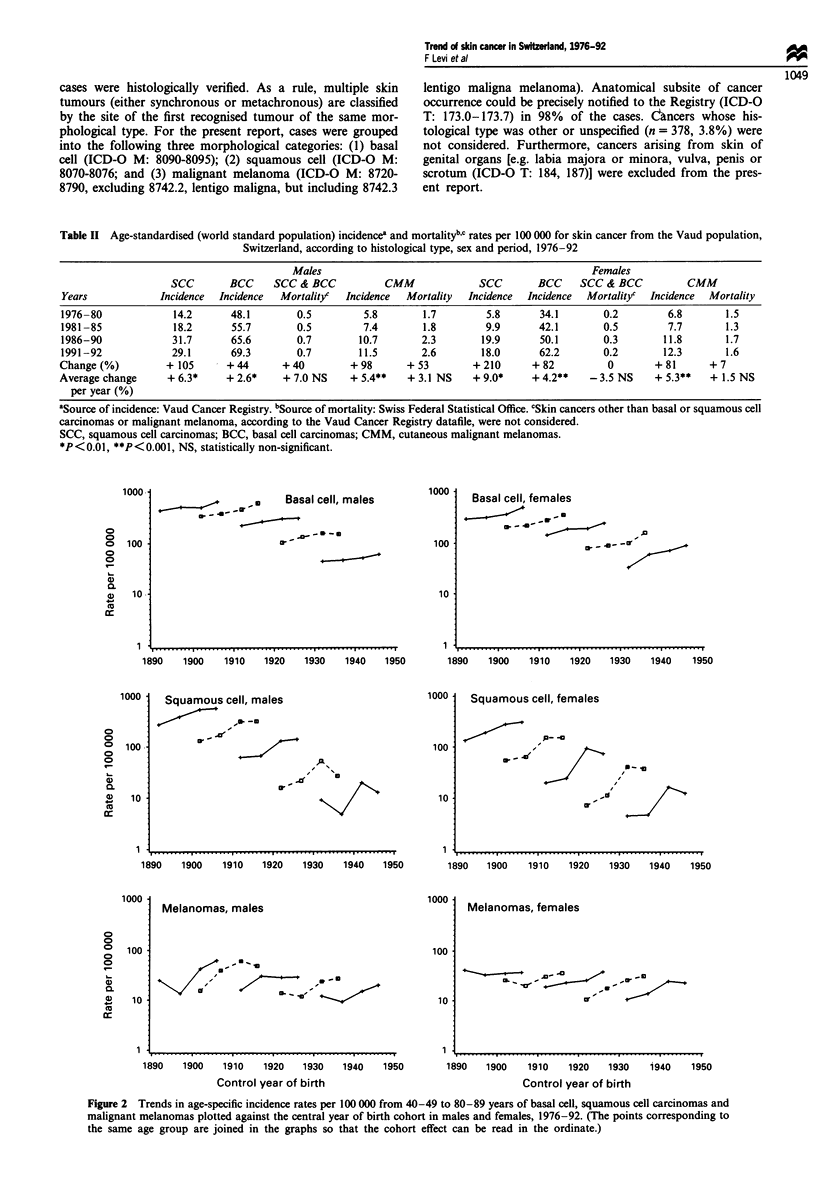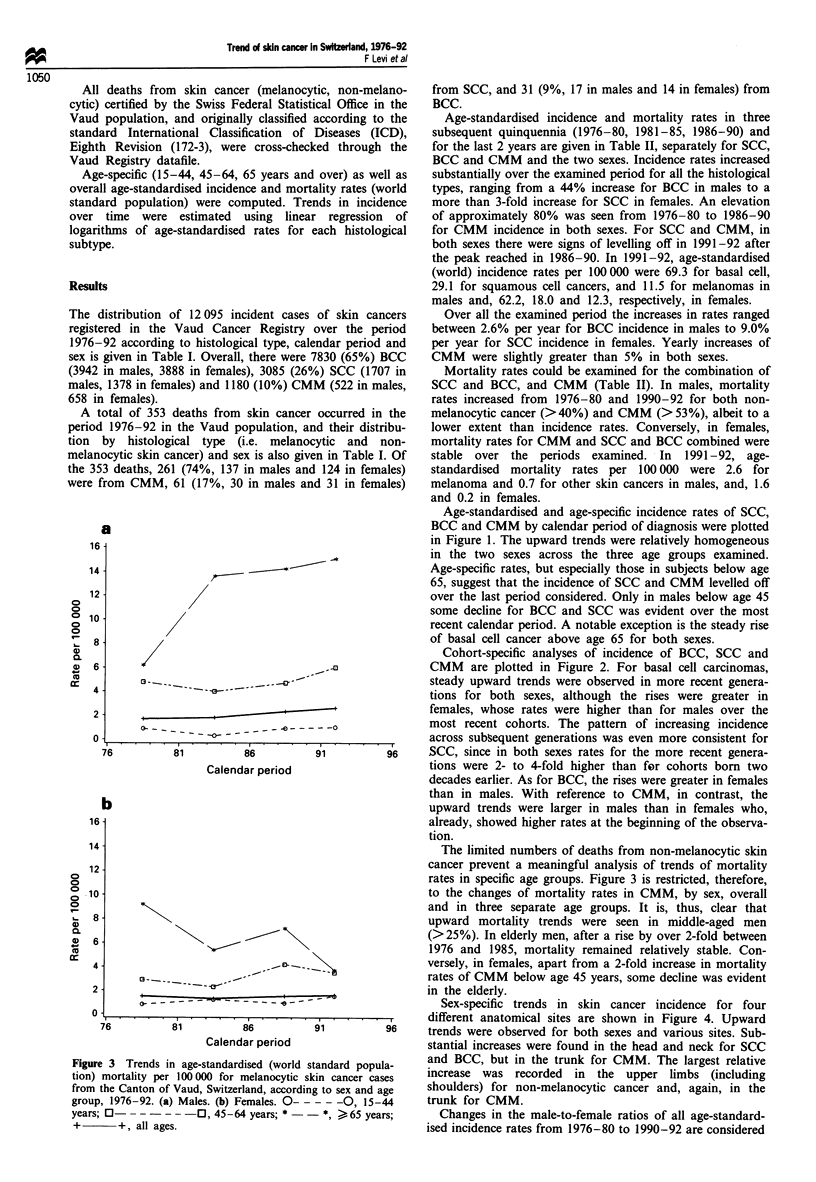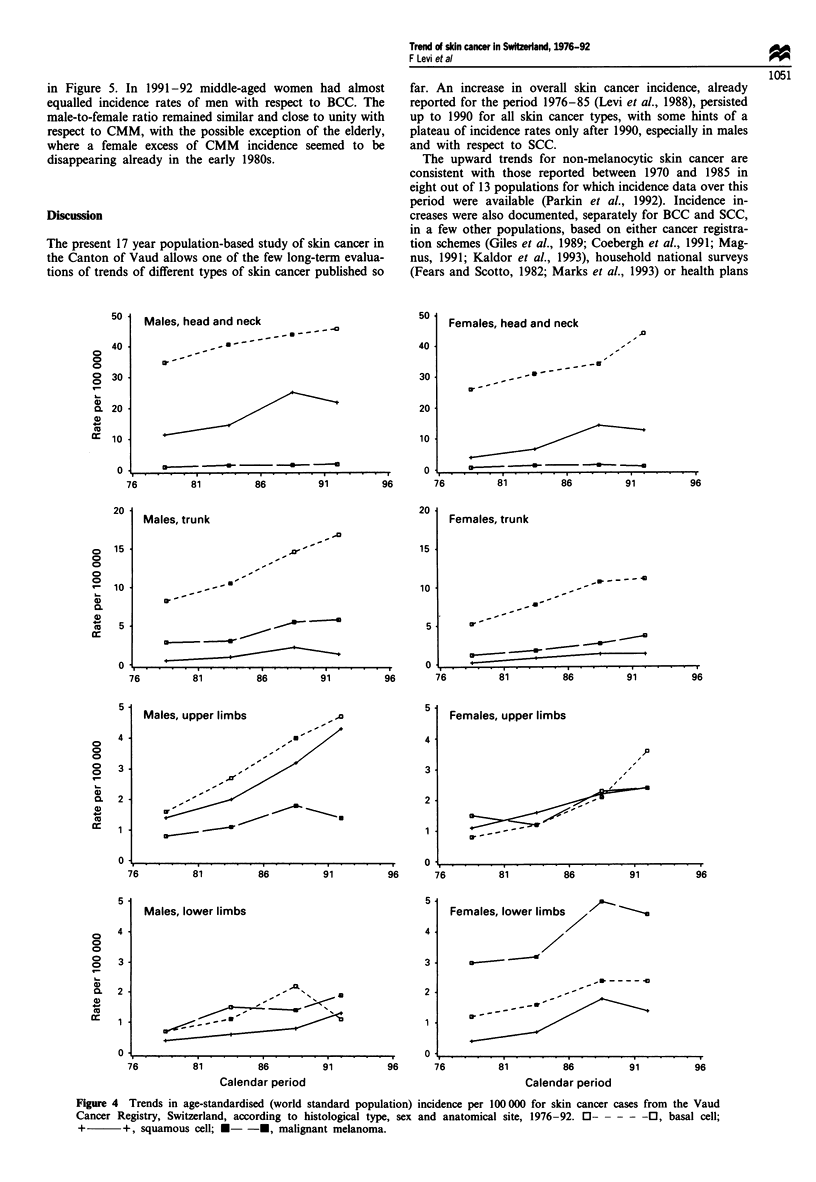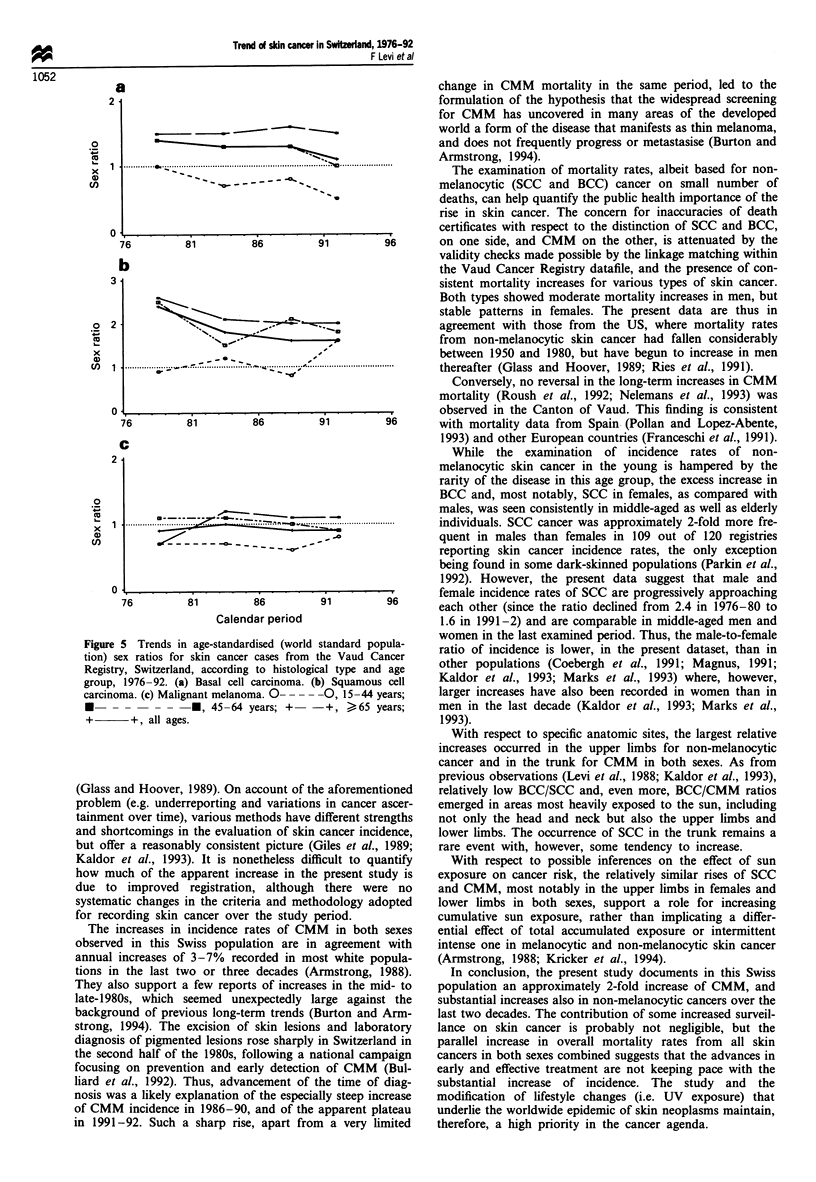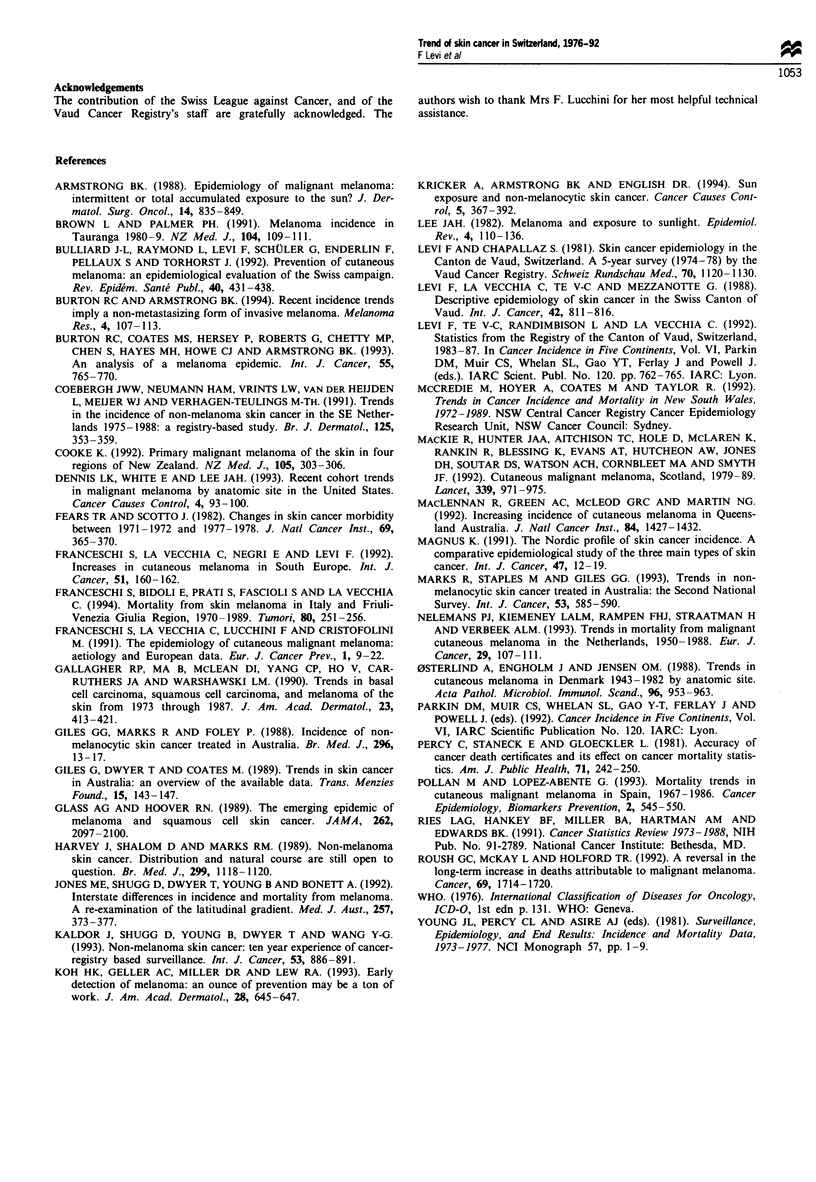# Trends of skin cancer in the Canton of Vaud, 1976-92.

**DOI:** 10.1038/bjc.1995.460

**Published:** 1995-10

**Authors:** F. Levi, S. Franceschi, V. C. Te, L. Randimbison, C. La Vecchia

**Affiliations:** Institut universitaire de médecine sociale et préventive, Centre Hospitalier Universitaire Vaudois, Lausanne, Switzerland.

## Abstract

Trends in incidence and mortality for basal cell carcinomas (BCC), squamous cell carcinomas (SCC) and cutaneous malignant melanoma (CMM) for the period 1976-92 were analysed using data from the Cancer Registry of the Swiss Canton of Vaud. Among the 12,473 cases registered, 63% were basal cell carcinomas, 25% squamous cell cancers, 9% cutaneous malignant melanomas and 3% other miscellaneous histological types. Age-standardised incidence rates increased substantially for all histological types considered, from 44% increase for BCC in males to a more than 3-fold increase for SCC in females, with only signs of a levelling off in 1991-92, following a peak of incidence rates in 1986-90. From 1976-80 to 1991-92 CMM incidence increased by approximately 80% in both sexes. In 1991-92, age-standardised (world) incidence rates per 100,000 were 69.3 for basal cell, 29.1 for squamous cell cancers and 11.5 for melanomas in males, and, respectively, 62.2, 18.0 and 12.3 in females. With respect to mortality, in males rates increased for both non-melanocytic cancer (> 40%) and CMM (> 53%) whereas in females CMM, BCC and SCC rates remained approximately stable over the calendar periods examined. In 1991-92, age-standardised mortality rates per 100,000 were 2.6 for melanoma and 0.7 for other skin cancers in males, and, respectively, 1.6 and 0.2 in females. Upward trends in incidence were also present, and relatively homogeneous across, various age groups examined. However, SCC and CMM levelled off over the last period, and some decline was apparent in males below age 45. Separate analysis by anatomical site showed substantial increases in the head and neck for SCC and BCC, and in the trunk for CMM. In 1991-92, middle-aged women had almost equalled male incidence rates of BCC and SCC. A female excess of CMM incidence seemed to have disappeared since 1981-86. The increase in skin cancer incidence thus continued in this population up to the late 1980s, with a plateau only after 1990.


British Journal of Cancer (1995) 72, 1047-1053
© 1995 Stockton Press All rights reserved 0007-0920/95 $12.00

### Trends of skin cancer in the Canton of Vaud, 1976-92

F Levi[1], S Franceschi[2], V-C Te[1], L Randimbison[1] and C La Vecchia[3]

[1]*Registre vaudois des tumeurs, Institut universitaire de médecine sociale et préventive, Centre Hospitalier Universitaire Vaudois, Falaises 1, 1011 Lausanne, Switzerland;* [2]*Servizio di Epidemiologia, Centro di Riferimento Oncologico, Via Pedemontana Occ, 33081 Aviano, Italy;* [3]*Istituto di Ricerche Farmacologiche 'Mario Negri', and Istituto di Statistica Medica e Biometria, Università di Milano, Via Venezian 1, 20133 Milan, Italy.*

**Summary** Trends in incidence and mortality for basal cell carcinomas (BCC), squamous cell carcinomas (SCC) and cutaneous malignant melanoma (CMM) for the period 1976-92 were analysed using data from the Cancer Registry of the Swiss Canton of Vaud. Among the 12 473 cases registered, 63% were basal cell carcinomas, 25% squamous cell cancers, 9% cutaneous malignant melanomas and 3% other miscellaneous histological types. Age-standardised incidence rates increased substantially for all histological types considered, from 44% increase for BCC in males to a more than 3-fold increase for SCC in females, with only signs of a levelling off in 1991-92, following a peak of incidence rates in 1986-90. From 1976-80 to 1991-92 CMM incidence increased by approximately 80% in both sexes. In 1991-92, age-standardised (world) incidence rates per 100 000 were 69.3 for basal cell, 29.1 for squamous cell cancers and 11.5 for melanomas in males, and, respectively, 62.2, 18.0 and 12.3 in females. With respect to mortality, in males rates increased for both non-melanocytic cancer (>40%) and CMM (>53%) whereas in females CMM, BCC and SCC rates remained approximately stable over the calendar periods examined. In 1991-92, age-standardised mortality rates per 100 000 were 2.6 for melanoma and 0.7 for other skin cancers in males, and, respectively, 1.6 and 0.2 in females. Upward trends in incidence were also present, and relatively homogeneous across, various age groups examined. However, SCC and CMM levelled off over the last period, and some decline was apparent in males below age 45. Separate analysis by anatomical site showed substantial increases in the head and neck for SCC and BCC, and in the trunk for CMM. In 1991-92, middle-aged women had almost equalled male incidence rates of BCC and SCC. A female excess of CMM incidence seemed to have disappeared since 1981-86. The increase in skin cancer incidence thus continued in this population up to the late 1980s, with a plateau only after 1990.

**Keywords**: skin; epidemiology; aetiology; incidence; mortality; time trends

The analysis and interpretation of temporal trends for skin cancer is hampered by difficulties in data collection and interpretation to a larger extent than for most other cancer sites or types. The first problem stems from the coexistence of at least three main histotypes of skin cancer; basal cell carcinoma (BCC), squamous cell carcinoma (SCC), and cutaneous malignant melanoma (CMM), substantially differing from each other from an epidemiological and clinical viewpoint. At the level of death certificates, however, the distinction between the three types of skin cancer is largely unsatisfactory (Percy *et al.*, 1981). Population-based sources of incidence also present weaknesses ranging from explicit exclusion of skin cancer from certain cancer registration schemes (Parkin *et al.*, 1992) to various degrees of under-notification (Koh *et al.*, 1993) and multiple registration (Young *et al.*, 1981). Finally, a large proportion of skin cancers, most notably BCC (Harvey *et al.*, 1989; Burton and Armstrong, 1994), is almost invariably non-fatal, and often treated with out-patient procedures. Incidence rates are thus likely to be heavily influenced by the extent to which people seek medical advice and the skin lesion undergoes surgical resection and histological examination. All these circumstances tend to exaggerate upward trends and, anyhow, make international comparisons difficult.

These factors notwithstanding, several reports of upward trends in mortality and incidence rates of CMM, BCC, SCC and other skin neoplasms have been published, especially from the Australasian Continent (Giles *et al.*, 1989; Brown and Palmer, 1991; Cooke, 1992; Jones *et al.*, 1992; McCredie *et al.*, 1992; MacLennan *et al.*, 1992; Burton *et al.*, 1993), the US (Glass and Hoover, 1989; Ries *et al.*, 1991; Dennis *et al.*, 1993), and Europe (Levi *et al.*, 1988; Osterlind *et al.*, 1988; Magnus, 1991; Mackie *et al.*, 1992; Nelemans *et al.*, 1993), including southern Europe (Franceschi *et al.*, 1992; Pollan and Lopez Abente, 1993; Franceschi *et al.*, 1994). Data on time trends in the occurrence of BCC and SCC, especially population-based data, are much scantier (Levi *et al.*, 1988; Gallagher *et al.*, 1990; Coebergh *et al.*, 1991; Magnus, 1991; Kaldor *et al.*, 1993; Marks *et al.*, 1993).

The present study took advantage of a population-based registration system which includes non-melanocytic skin cancer and has been operating since 1976. It was, therefore, possible to update a published population-based series of skin cancer cases (Levi *et al.*, 1988) up to 1992. A particularly favourable environment for skin cancer registration has long been present in this population, since in this region, traditionally, the large majority of cutaneous lesions surgically resected are examined by a pathologist. Thus, previous reports from this series (Levi and Chapallaz, 1981; Levi *et al.*, 1988) have been frequently quoted as standard reference figures (Lee, 1982; Giles *et al.*, 1988). This allows a detailed analysis of time trends of both incidence and mortality of BCC, SCC and CMM, including separate analyses of sexes, various age groups and anatomical subsites.

### Materials and methods

The data were abstracted from the Vaud Cancer Registry file, which includes information concerning incident cases of malignant neoplasms in the Canton (whose population, according to the 1990 Census, was about 580 000 inhabitants; Levi *et al.*, 1992). Information collected by the register includes general demographic characteristics of the patient (age, sex, municipality of residence), site and histological type of the tumour according to the standard International Classification of Diseases for Oncology (WHO, 1976), and time of diagnostic confirmation.

The present series comprises 12 095 incident skin cancer primaries (6171 males and 5924 females) registered from 1976 to 1992 in the population of the Swiss Canton of Vaud. All

Correspondence: F Levi
Received 27 September 1994; revised 10 April 1995; accepted 15 May 1995.

**Table I** Distribution of 12 095 incident cases[a] and 353 deaths[b,c] from skin cancer in Vaud, Switzerland, according to histological type, calendar period and sex, 1976-92

| | *Males* | | | | | | *Females* | | | | | |
|---|---|---|---|---|---|---|---|---|---|---|---|---|
| | *Incident cases* | | | | | *Deaths* | *Incident cases* | | | | | *Deaths* |
| *Histological type* | *1976-80* | *1981-85* | *1986-90* | *1991-92* | *Total* | *total* | *1976-80* | *1981-85* | *1986-90* | *1991-92* | *Total* | *total* |
| Basal-cell carcinoma | 893 | 1086 | 1350 | 613 | 3942 | 17 | 817 | 1042 | 1339 | 690 | 3888 | 14 |
| Squamous-cell carcinoma | 288 | 399 | 733 | 287 | 1707 | 30 | 175 | 315 | 634 | 254 | 1378 | 31 |
| Cutaneous malignant melanoma | 96 | 133 | 200 | 93 | 522 | 137 | 138 | 160 | 253 | 107 | 658 | 124 |
| Total | 1277 | 1618 | 2283 | 993 | 6171 | 184 | 1130 | 1517 | 2226 | 1051 | 5924 | 169 |

[a]Source of incidence data: Vaud Cancer Registry (378 skin cancer cases whose histological type was other or unspecified were not considered). [b]Source of mortality data: Swiss Federal Statistical Office. [c]Skin cancers other than basal- or squamous-cell carcinomas, and malignant melanoma, according to the Vaud Cancer Registry datafile, were not considered.

**Figure 1** Trends in age-standardised (world standard population) incidence per 100 000 for skin cancer cases from the Vaud Cancer Registry, Switzerland, according to histological type, sex and age group, 1976-92. □- - - - -□, basal cell; +———+, squamous cell; ■— —■, malignant melanoma.

cases were histologically verified. As a rule, multiple skin tumours (either synchronous or metachronous) are classified by the site of the first recognised tumour of the same morphological type. For the present report, cases were grouped into the following three morphological categories: (1) basal cell (ICD-O M: 8090-8095); (2) squamous cell (ICD-O M: 8070-8076; and (3) malignant melanoma (ICD-O M: 8720-8790, excluding 8742.2, lentigo maligna, but including 8742.3 lentigo maligna melanoma). Anatomical subsite of cancer occurrence could be precisely notified to the Registry (ICD-O T: 173.0-173.7) in 98% of the cases. Cancers whose histological type was other or unspecified ($n = 378$, 3.8%) were not considered. Furthermore, cancers arising from skin of genital organs [e.g. labia majora or minora, vulva, penis or scrotum (ICD-O T: 184, 187)] were excluded from the present report.

**Table II** Age-standardised (world standard population) incidence[a] and mortality[b,c] rates per 100 000 for skin cancer from the Vaud population, Switzerland, according to histological type, sex and period, 1976-92

| | *Males* | | | | | *Females* | | | | |
|---|---|---|---|---|---|---|---|---|---|---|
| *Years* | *SCC Incidence* | *BCC Incidence* | *SCC & BCC Mortality*[c] | *CMM Incidence* | *CMM Mortality* | *SCC Incidence* | *BCC Incidence* | *SCC & BCC Mortality*[c] | *CMM Incidence* | *CMM Mortality* |
| 1976-80 | 14.2 | 48.1 | 0.5 | 5.8 | 1.7 | 5.8 | 34.1 | 0.2 | 6.8 | 1.5 |
| 1981-85 | 18.2 | 55.7 | 0.5 | 7.4 | 1.8 | 9.9 | 42.1 | 0.5 | 7.7 | 1.3 |
| 1986-90 | 31.7 | 65.6 | 0.7 | 10.7 | 2.3 | 19.9 | 50.1 | 0.3 | 11.8 | 1.7 |
| 1991-92 | 29.1 | 69.3 | 0.7 | 11.5 | 2.6 | 18.0 | 62.2 | 0.2 | 12.3 | 1.6 |
| Change (%) | + 105 | + 44 | + 40 | + 98 | + 53 | + 210 | + 82 | 0 | + 81 | + 7 |
| Average change per year (%) | + 6.3* | + 2.6* | + 7.0 NS | + 5.4** | + 3.1 NS | + 9.0* | + 4.2** | - 3.5 NS | + 5.3** | + 1.5 NS |

[a]Source of incidence: Vaud Cancer Registry. [b]Source of mortality: Swiss Federal Statistical Office. [c]Skin cancers other than basal or squamous cell carcinomas or malignant melanoma, according to the Vaud Cancer Registry datafile, were not considered.
SCC, squamous cell carcinomas; BCC, basal cell carcinomas; CMM, cutaneous malignant melanomas.
*$P<0.01$, **$P<0.001$, NS, statistically non-significant.

**Figure 2** Trends in age-specific incidence rates per 100 000 from 40-49 to 80-89 years of basal cell, squamous cell carcinomas and malignant melanomas plotted against the central year of birth cohort in males and females, 1976-92. (The points corresponding to the same age group are joined in the graphs so that the cohort effect can be read in the ordinate.)

All deaths from skin cancer (melanocytic, non-melanocytic) certified by the Swiss Federal Statistical Office in the Vaud population, and originally classified according to the standard International Classification of Diseases (ICD), Eighth Revision (172-3), were cross-checked through the Vaud Registry datafile.

Age-specific (15–44, 45–64, 65 years and over) as well as overall age-standardised incidence and mortality rates (world standard population) were computed. Trends in incidence over time were estimated using linear regression of logarithms of age-standardised rates for each histological subtype.

### Results

The distribution of 12 095 incident cases of skin cancers registered in the Vaud Cancer Registry over the period 1976–92 according to histological type, calendar period and sex is given in Table I. Overall, there were 7830 (65%) BCC (3942 in males, 3888 in females), 3085 (26%) SCC (1707 in males, 1378 in females) and 1180 (10%) CMM (522 in males, 658 in females).

A total of 353 deaths from skin cancer occurred in the period 1976–92 in the Vaud population, and their distribution by histological type (i.e. melanocytic and non-melanocytic skin cancer) and sex is also given in Table I. Of the 353 deaths, 261 (74%, 137 in males and 124 in females) were from CMM, 61 (17%, 30 in males and 31 in females) from SCC, and 31 (9%, 17 in males and 14 in females) from BCC.

**Figure 3** Trends in age-standardised (world standard population) mortality per 100 000 for melanocytic skin cancer cases from the Canton of Vaud, Switzerland, according to sex and age group, 1976–92. (**a**) Males. (**b**) Females. O- - - - -O, 15–44 years; □— — — — — — — —□, 45–64 years; * — — *, ≥65 years; +———+, all ages.

Age-standardised incidence and mortality rates in three subsequent quinquennia (1976–80, 1981–85, 1986–90) and for the last 2 years are given in Table II, separately for SCC, BCC and CMM and the two sexes. Incidence rates increased substantially over the examined period for all the histological types, ranging from a 44% increase for BCC in males to a more than 3-fold increase for SCC in females. An elevation of approximately 80% was seen from 1976–80 to 1986–90 for CMM incidence in both sexes. For SCC and CMM, in both sexes there were signs of levelling off in 1991–92 after the peak reached in 1986–90. In 1991–92, age-standardised (world) incidence rates per 100 000 were 69.3 for basal cell, 29.1 for squamous cell cancers, and 11.5 for melanomas in males and, 62.2, 18.0 and 12.3, respectively, in females.

Over all the examined period the increases in rates ranged between 2.6% per year for BCC incidence in males to 9.0% per year for SCC incidence in females. Yearly increases of CMM were slightly greater than 5% in both sexes.

Mortality rates could be examined for the combination of SCC and BCC, and CMM (Table II). In males, mortality rates increased from 1976–80 and 1990–92 for both non-melanocytic cancer (>40%) and CMM (>53%), albeit to a lower extent than incidence rates. Conversely, in females, mortality rates for CMM and SCC and BCC combined were stable over the periods examined. In 1991–92, age-standardised mortality rates per 100 000 were 2.6 for melanoma and 0.7 for other skin cancers in males, and, 1.6 and 0.2 in females.

Age-standardised and age-specific incidence rates of SCC, BCC and CMM by calendar period of diagnosis were plotted in Figure 1. The upward trends were relatively homogeneous in the two sexes across the three age groups examined. Age-specific rates, but especially those in subjects below age 65, suggest that the incidence of SCC and CMM levelled off over the last period considered. Only in males below age 45 some decline for BCC and SCC was evident over the most recent calendar period. A notable exception is the steady rise of basal cell cancer above age 65 for both sexes.

Cohort-specific analyses of incidence of BCC, SCC and CMM are plotted in Figure 2. For basal cell carcinomas, steady upward trends were observed in more recent generations for both sexes, although the rises were greater in females, whose rates were higher than for males over the most recent cohorts. The pattern of increasing incidence across subsequent generations was even more consistent for SCC, since in both sexes rates for the more recent generations were 2- to 4-fold higher than for cohorts born two decades earlier. As for BCC, the rises were greater in females than in males. With reference to CMM, in contrast, the upward trends were larger in males than in females who, already, showed higher rates at the beginning of the observation.

The limited numbers of deaths from non-melanocytic skin cancer prevent a meaningful analysis of trends of mortality rates in specific age groups. Figure 3 is restricted, therefore, to the changes of mortality rates in CMM, by sex, overall and in three separate age groups. It is, thus, clear that upward mortality trends were seen in middle-aged men (>25%). In elderly men, after a rise by over 2-fold between 1976 and 1985, mortality remained relatively stable. Conversely, in females, apart from a 2-fold increase in mortality rates of CMM below age 45 years, some decline was evident in the elderly.

Sex-specific trends in skin cancer incidence for four different anatomical sites are shown in Figure 4. Upward trends were observed for both sexes and various sites. Substantial increases were found in the head and neck for SCC and BCC, but in the trunk for CMM. The largest relative increase was recorded in the upper limbs (including shoulders) for non-melanocytic cancer and, again, in the trunk for CMM.

Changes in the male-to-female ratios of all age-standardised incidence rates from 1976–80 to 1990–92 are considered in Figure 5. In 1991-92 middle-aged women had almost equalled incidence rates of men with respect to BCC. The male-to-female ratio remained similar and close to unity with respect to CMM, with the possible exception of the elderly, where a female excess of CMM incidence seemed to be disappearing already in the early 1980s.

### Discussion

The present 17 year population-based study of skin cancer in the Canton of Vaud allows one of the few long-term evaluations of trends of different types of skin cancer published so far. An increase in overall skin cancer incidence, already reported for the period 1976-85 (Levi *et al.*, 1988), persisted up to 1990 for all skin cancer types, with some hints of a plateau of incidence rates only after 1990, especially in males and with respect to SCC.

The upward trends for non-melanocytic skin cancer are consistent with those reported between 1970 and 1985 in eight out of 13 populations for which incidence data over this period were available (Parkin *et al.*, 1992). Incidence increases were also documented, separately for BCC and SCC, in a few other populations, based on either cancer registration schemes (Giles *et al.*, 1989; Coebergh *et al.*, 1991; Magnus, 1991; Kaldor *et al.*, 1993), household national surveys (Fears and Scotto, 1982; Marks *et al.*, 1993) or health plans

**Figure 4** Trends in age-standardised (world standard population) incidence per 100 000 for skin cancer cases from the Vaud Cancer Registry, Switzerland, according to histological type, sex and anatomical site, 1976-92. □- - - - -□, basal cell; +———+, squamous cell; ■— —■, malignant melanoma.

**Figure 5** Trends in age-standardised (world standard population) sex ratios for skin cancer cases from the Vaud Cancer Registry, Switzerland, according to histological type and age group, 1976–92. (**a**) Basal cell carcinoma. (**b**) Squamous cell carcinoma. (**c**) Malignant melanoma. O- - - - -O, 15–44 years; ■— — — — — —■, 45–64 years; +— —+, ⩾65 years; +———+, all ages.

(Glass and Hoover, 1989). On account of the aforementioned problem (e.g. underreporting and variations in cancer ascertainment over time), various methods have different strengths and shortcomings in the evaluation of skin cancer incidence, but offer a reasonably consistent picture (Giles *et al.*, 1989; Kaldor *et al.*, 1993). It is nonetheless difficult to quantify how much of the apparent increase in the present study is due to improved registration, although there were no systematic changes in the criteria and methodology adopted for recording skin cancer over the study period.

The increases in incidence rates of CMM in both sexes observed in this Swiss population are in agreement with annual increases of 3–7% recorded in most white populations in the last two or three decades (Armstrong, 1988). They also support a few reports of increases in the mid- to late-1980s, which seemed unexpectedly large against the background of previous long-term trends (Burton and Armstrong, 1994). The excision of skin lesions and laboratory diagnosis of pigmented lesions rose sharply in Switzerland in the second half of the 1980s, following a national campaign focusing on prevention and early detection of CMM (Bulliard *et al.*, 1992). Thus, advancement of the time of diagnosis was a likely explanation of the especially steep increase of CMM incidence in 1986–90, and of the apparent plateau in 1991–92. Such a sharp rise, apart from a very limited change in CMM mortality in the same period, led to the formulation of the hypothesis that the widespread screening for CMM has uncovered in many areas of the developed world a form of the disease that manifests as thin melanoma, and does not frequently progress or metastasise (Burton and Armstrong, 1994).

The examination of mortality rates, albeit based for non-melanocytic (SCC and BCC) cancer on small number of deaths, can help quantify the public health importance of the rise in skin cancer. The concern for inaccuracies of death certificates with respect to the distinction of SCC and BCC, on one side, and CMM on the other, is attenuated by the validity checks made possible by the linkage matching within the Vaud Cancer Registry datafile, and the presence of consistent mortality increases for various types of skin cancer. Both types showed moderate mortality increases in men, but stable patterns in females. The present data are thus in agreement with those from the US, where mortality rates from non-melanocytic skin cancer had fallen considerably between 1950 and 1980, but have begun to increase in men thereafter (Glass and Hoover, 1989; Ries *et al.*, 1991).

Conversely, no reversal in the long-term increases in CMM mortality (Roush *et al.*, 1992; Nelemans *et al.*, 1993) was observed in the Canton of Vaud. This finding is consistent with mortality data from Spain (Pollan and Lopez-Abente, 1993) and other European countries (Franceschi *et al.*, 1991).

While the examination of incidence rates of non-melanocytic skin cancer in the young is hampered by the rarity of the disease in this age group, the excess increase in BCC and, most notably, SCC in females, as compared with males, was seen consistently in middle-aged as well as elderly individuals. SCC cancer was approximately 2-fold more frequent in males than females in 109 out of 120 registries reporting skin cancer incidence rates, the only exception being found in some dark-skinned populations (Parkin *et al.*, 1992). However, the present data suggest that male and female incidence rates of SCC are progressively approaching each other (since the ratio declined from 2.4 in 1976–80 to 1.6 in 1991–2) and are comparable in middle-aged men and women in the last examined period. Thus, the male-to-female ratio of incidence is lower, in the present dataset, than in other populations (Coebergh *et al.*, 1991; Magnus, 1991; Kaldor *et al.*, 1993; Marks *et al.*, 1993) where, however, larger increases have also been recorded in women than in men in the last decade (Kaldor *et al.*, 1993; Marks *et al.*, 1993).

With respect to specific anatomic sites, the largest relative increases occurred in the upper limbs for non-melanocytic cancer and in the trunk for CMM in both sexes. As from previous observations (Levi *et al.*, 1988; Kaldor *et al.*, 1993), relatively low BCC/SCC and, even more, BCC/CMM ratios emerged in areas most heavily exposed to the sun, including not only the head and neck but also the upper limbs and lower limbs. The occurrence of SCC in the trunk remains a rare event with, however, some tendency to increase.

With respect to possible inferences on the effect of sun exposure on cancer risk, the relatively similar rises of SCC and CMM, most notably in the upper limbs in females and lower limbs in both sexes, support a role for increasing cumulative sun exposure, rather than implicating a differential effect of total accumulated exposure or intermittent intense one in melanocytic and non-melanocytic skin cancer (Armstrong, 1988; Kricker *et al.*, 1994).

In conclusion, the present study documents in this Swiss population an approximately 2-fold increase of CMM, and substantial increases also in non-melanocytic cancers over the last two decades. The contribution of some increased surveillance on skin cancer is probably not negligible, but the parallel increase in overall mortality rates from all skin cancers in both sexes combined suggests that the advances in early and effective treatment are not keeping pace with the substantial increase of incidence. The study and the modification of lifestyle changes (i.e. UV exposure) that underlie the worldwide epidemic of skin neoplasms maintain, therefore, a high priority in the cancer agenda.

**Acknowledgements**
The contribution of the Swiss League against Cancer, and of the Vaud Cancer Registry's staff are gratefully acknowledged. The authors wish to thank Mrs F. Lucchini for her most helpful technical assistance.

**References**

ARMSTRONG BK. (1988). Epidemiology of malignant melanoma: intermittent or total accumulated exposure to the sun? *J. Dermatol. Surg. Oncol.*, **14,** 835-849.

BROWN L AND PALMER PH. (1991). Melanoma incidence in Tauranga 1980-9. *NZ Med. J.*, **104,** 109-111.

BULLIARD J-L, RAYMOND L, LEVI F, SCHÜLER G, ENDERLIN F, PELLAUX S AND TORHORST J. (1992). Prevention of cutaneous melanoma: an epidemiological evaluation of the Swiss campaign. *Rev. Epidém. Santé Publ.*, **40,** 431-438.

BURTON RC AND ARMSTRONG BK. (1994). Recent incidence trends imply a non-metastasizing form of invasive melanoma. *Melanoma Res.*, **4,** 107-113.

BURTON RC, COATES MS, HERSEY P, ROBERTS G, CHETTY MP, CHEN S, HAYES MH, HOWE CJ AND ARMSTRONG BK. (1993). An analysis of a melanoma epidemic. *Int. J. Cancer*, **55,** 765-770.

COEBERGH JWW, NEUMANN HAM, VRINTS LW, VAN DER HEIJDEN L, MEIJER WJ AND VERHAGEN-TEULINGS M-TH. (1991). Trends in the incidence of non-melanoma skin cancer in the SE Netherlands 1975-1988: a registry-based study. *Br. J. Dermatol.*, **125,** 353-359.

COOKE K. (1992). Primary malignant melanoma of the skin in four regions of New Zealand. *NZ Med. J.*, **105,** 303-306.

DENNIS LK, WHITE E AND LEE JAH. (1993). Recent cohort trends in malignant melanoma by anatomic site in the United States. *Cancer Causes Control*, **4,** 93-100.

FEARS TR AND SCOTTO J. (1982). Changes in skin cancer morbidity between 1971-1972 and 1977-1978. *J. Natl Cancer Inst.*, **69,** 365-370.

FRANCESCHI S, LA VECCHIA C, NEGRI E AND LEVI F. (1992). Increases in cutaneous melanoma in South Europe. *Int. J. Cancer*, **51,** 160-162.

FRANCESCHI S, BIDOLI E, PRATI S, FASCIOLI S AND LA VECCHIA C. (1994). Mortality from skin melanoma in Italy and Friuli-Venezia Giulia Region, 1970-1989. *Tumori*, **80,** 251-256.

FRANCESCHI S, LA VECCHIA C, LUCCHINI F AND CRISTOFOLINI M. (1991). The epidemiology of cutaneous malignant melanoma: aetiology and European data. *Eur. J. Cancer Prev.*, **1,** 9-22.

GALLAGHER RP, MA B, MCLEAN DI, YANG CP, HO V, CARRUTHERS JA AND WARSHAWSKI LM. (1990). Trends in basal cell carcinoma, squamous cell carcinoma, and melanoma of the skin from 1973 through 1987. *J. Am. Acad. Dermatol.*, **23,** 413-421.

GILES GG, MARKS R AND FOLEY P. (1988). Incidence of non-melanocytic skin cancer treated in Australia. *Br. Med. J.*, **296,** 13-17.

GILES G, DWYER T AND COATES M. (1989). Trends in skin cancer in Australia: an overview of the available data. *Trans. Menzies Found.*, **15,** 143-147.

GLASS AG AND HOOVER RN. (1989). The emerging epidemic of melanoma and squamous cell skin cancer. *JAMA*, **262,** 2097-2100.

HARVEY J, SHALOM D AND MARKS RM. (1989). Non-melanoma skin cancer. Distribution and natural course are still open to question. *Br. Med. J.*, **299,** 1118-1120.

JONES ME, SHUGG D, DWYER T, YOUNG B AND BONETT A. (1992). Interstate differences in incidence and mortality from melanoma. A re-examination of the latitudinal gradient. *Med. J. Aust.*, **257,** 373-377.

KALDOR J, SHUGG D, YOUNG B, DWYER T AND WANG Y-G. (1993). Non-melanoma skin cancer: ten year experience of cancer-registry based surveillance. *Int. J. Cancer*, **53,** 886-891.

KOH HK, GELLER AC, MILLER DR AND LEW RA. (1993). Early detection of melanoma: an ounce of prevention may be a ton of work. *J. Am. Acad. Dermatol.*, **28,** 645-647.

KRICKER A, ARMSTRONG BK AND ENGLISH DR. (1994). Sun exposure and non-melanocytic skin cancer. *Cancer Causes Control*, **5,** 367-392.

LEE JAH. (1982). Melanoma and exposure to sunlight. *Epidemiol. Rev.*, **4,** 110-136.

LEVI F AND CHAPALLAZ S. (1981). Skin cancer epidemiology in the Canton de Vaud, Switzerland. A 5-year survey (1974-78) by the Vaud Cancer Registry. *Schweiz Rundschau Med.*, **70,** 1120-1130.

LEVI F, LA VECCHIA C, TE V-C AND MEZZANOTTE G. (1988). Descriptive epidemiology of skin cancer in the Swiss Canton of Vaud. *Int. J. Cancer*, **42,** 811-816.

LEVI F, TE V-C, RANDIMBISON L AND LA VECCHIA C. (1992). Statistics from the Registry of the Canton of Vaud, Switzerland, 1983-87. In *Cancer Incidence in Five Continents*, Vol. VI, Parkin DM, Muir CS, Whelan SL, Gao YT, Ferlay J and Powell J. (eds.). IARC Scient. Publ. No. 120. pp. 762-765. IARC: Lyon.

MCCREDIE M, HOYER A, COATES M AND TAYLOR R. (1992). *Trends in Cancer Incidence and Mortality in New South Wales, 1972-1989*. NSW Central Cancer Registry Cancer Epidemiology Research Unit, NSW Cancer Council: Sydney.

MACKIE R, HUNTER JAA, AITCHISON TC, HOLE D, MCLAREN K, RANKIN R, BLESSING K, EVANS AT, HUTCHEON AW, JONES DH, SOUTAR DS, WATSON ACH, CORNBLEET MA AND SMYTH JF. (1992). Cutaneous malignant melanoma, Scotland, 1979-89. *Lancet*, **339,** 971-975.

MACLENNAN R, GREEN AC, MCLEOD GRC AND MARTIN NG. (1992). Increasing incidence of cutaneous melanoma in Queensland Australia. *J. Natl Cancer Inst.*, **84,** 1427-1432.

MAGNUS K. (1991). The Nordic profile of skin cancer incidence. A comparative epidemiological study of the three main types of skin cancer. *Int. J. Cancer*, **47,** 12-19.

MARKS R, STAPLES M AND GILES GG. (1993), Trends in non-melanocytic skin cancer treated in Australia: the Second National Survey. *Int. J. Cancer*, **53,** 585-590.

NELEMANS PJ, KIEMENEY LALM, RAMPEN FHJ, STRAATMAN H AND VERBEEK ALM. (1993). Trends in mortality from malignant cutaneous melanoma in the Netherlands, 1950-1988. *Eur. J. Cancer*, **29,** 107-111.

ØSTERLIND A, ENGHOLM J AND JENSEN OM. (1988). Trends in cutaneous melanoma in Denmark 1943-1982 by anatomic site. *Acta Pathol. Microbiol. Immunol. Scand.*, **96,** 953-963.

PARKIN DM, MUIR CS, WHELAN SL, GAO Y-T, FERLAY J AND POWELL J. (eds). (1992). *Cancer Incidence in Five Continents*, Vol. VI, IARC Scientific Publication No. 120. IARC: Lyon.

PERCY C, STANECK E AND GLOECKLER L. (1981). Accuracy of cancer death certificates and its effect on cancer mortality statistics. *Am. J. Public Health*, **71,** 242-250.

POLLAN M AND LOPEZ-ABENTE G. (1993). Mortality trends in cutaneous malignant melanoma in Spain, 1967-1986. *Cancer Epidemiology, Biomarkers Prevention*, **2,** 545-550.

RIES LAG, HANKEY BF, MILLER BA, HARTMAN AM AND EDWARDS BK. (1991). *Cancer Statistics Review 1973-1988*, NIH Pub. No. 91-2789. National Cancer Institute: Bethesda, MD.

ROUSH GC, MCKAY L AND HOLFORD TR. (1992). A reversal in the long-term increase in deaths attributable to malignant melanoma. *Cancer*, **69,** 1714-1720.

WHO. (1976). *International Classification of Diseases for Oncology, ICD-O*, 1st edn p. 131. WHO: Geneva.

YOUNG JL, PERCY CL AND ASIRE AJ (eds). (1981). *Surveillance, Epidemiology, and End Results: Incidence and Mortality Data, 1973-1977*. NCI Monograph 57, pp. 1-9.